# Structural core of the executive control network: A high angular resolution diffusion MRI study

**DOI:** 10.1002/hbm.24870

**Published:** 2019-11-25

**Authors:** Kai‐kai Shen, Thomas Welton, Matthew Lyon, Andrew N. McCorkindale, Greg T. Sutherland, Samantha Burnham, Jurgen Fripp, Ralph Martins, Stuart M. Grieve

**Affiliations:** ^1^ Australian eHealth Research Centre CSIRO Floreat Western Australia Australia; ^2^ Department of Biomedical Sciences Macquarie University Sydney New South Wales Australia; ^3^ Sydney Translational Imaging Laboratory Heart Research Institute, Charles Perkins Centre, University of Sydney Sydney New South Wales Australia; ^4^ Faculty of Medicine and Health Charles Perkins Centre and School of Medical Sciences, University of Sydney Sydney New South Wales Australia; ^5^ Department of Radiology Royal Prince Alfred Hospital Sydney New South Wales Australia

## Abstract

Executive function (EF) is a set of cognitive capabilities considered essential for successful daily living, and is negatively affected by ageing and neurodegenerative conditions. Underpinning EF performance are functional nodes in the executive control network (ECN), while the structural connectivity underlying this network is not well understood. In this paper, we evaluated the structural white matter tracts that interconnect the ECN and investigated their relationship to the EF performance. Using high‐angular resolution diffusion MRI data, we performed tractography analysis of structural connectivity in a cognitively normal cohort (*n* = 140), specifically targeting the connectivity between ECN nodes. Our data revealed the presence of a strongly‐connected “structural core” of the ECN comprising three components: interhemispheric frontal connections, a fronto‐parietal subnetwork and fronto‐striatal connections between right dorsolateral prefrontal cortex and right caudate. These pathways were strongly correlated with EF performance (*p* = .003). Post‐hoc analysis of subregions within the significant ECN connections showed that these effects were driven by a highly specific subset of interconnected cortical regions. The structural core subnetwork of the functional ECN may be an important feature crucial to a better future understanding of human cognition and behaviour.

## 1 INTRODUCTION

Executive function (EF) outlines a central set of cognitive capabilities that are essential to successful daily living. EF refers to a collection of higher order processes that guide thoughts and behaviours towards achieving a specific goal (Niendam et al., 2012). It includes processes such as working memory, inhibition of prepotent responses, attentional control, planning and flexibility of switching between different goals. Deficits in EF are observed across many brain diseases such as schizophrenia, major depressive disorder and Alzheimer's disease (Guarino et al., 2018; Snyder, 2013; Vohringer et al., 2013), and EF performance has been demonstrated to be an important factor influencing outcomes across many medical conditions including chronic cardiovascular and metabolic conditions (Broadley, White, & Andrew, 2017; Eggermont et al., 2012; C. Vincent & Hall, 2015). EF contributes to the coordination of activities across a wide range of cortical and subcortical brain structures that would make them vulnerable to reduced communication efficiency. Investigations into EF using functional MRI (fMRI) experiments have identified the activation patterns of an executive control network (ECN) subserving EF tasks (Niendam et al., 2012). However, the structural circuitry defining and moderating this complex function has not been described. It is therefore critical to develop a better understanding of the neural substrates of EF, including the ECN.

EF is primarily associated with the frontal lobe (Stuss & Alexander, 2000). Our previous work demonstrated a common network in various psychiatric conditions using voxel based morphometry analyses (Goodkind et al., 2015), highlighting an anterior insula/dorsal anterior cingulate‐based network which may relate to EF deficits. Following the identification of the ECN in task‐based fMRI studies (Nee et al., 2013; Niendam et al., 2012; Osaka et al., 2004; Taylor et al., 2004), it was also isolated as an intrinsic connectivity network in resting‐state fMRI studies (Beckmann, DeLuca, Devlin, & Smith, 2005; Damoiseaux et al., 2006; J. L. Vincent, Kahn, Snyder, Raichle, & Buckner, 2008), in which the correlations between BOLD signals arising from regions across the brain reveal patterns of dissociable networks (Seeley et al., 2007).

The patterns associated with EF are broadly distributed. The most commonly described components of the ECN are the prefrontal cortex, frontopolar cortex, anterior cingulate cortex and posterior parietal cortex (Damoiseaux et al., 2006). In addition to the frontal–parietal network, it is now recognised that the cuneus, supplementary motor area, motor‐related nodes, cingulo‐opercular nodes are also involved (Reineberg & Banich, 2016). Other studies have also identified EF‐related activities in the cerebellum and subcortical nuclei (Habas et al., 2009; Monchi, Petrides, Petre, Worsley, & Dagher, 2001). Shirer and colleagues (Shirer, Ryali, Rykhlevskaia, Menon, & Greicius, 2012) delineated the left and right ECN, consisting mainly of networks between dorsolateral prefrontal (dlPFC) and parietal cortices. The left ECN includes nodes in the left middle and superior frontal gyri, inferior frontal and orbitofrontal gyri, superior and inferior parietal, angular gyri, precuneus, inferior and middle temporal gyri, left thalamus and right crus. In the right ECN, there are nodes located in the right middle and superior frontal gyri, right inferior parietal, supramarginal, and angular gyri, left crus, and right caudate.

Very few diffusion MRI (dMRI) studies have specifically examined the structural WM connections within the ECN. It is often assumed that the functional connectivity within the ECN is likely to reflect underlying structural connectivity. Support for this finding comes from a small number of studies that have demonstrated strong correlation between fMRI and structural networks identified by dMRI (Damoiseaux & Greicius, 2009; Hagmann et al., 2008; Honey et al., 2009; Skudlarski et al., 2008). We demonstrated that poorer EF was associated with decreased WM integrity in the prefrontal cortex, parietal lobe and thalamic projections (Grieve, Williams, Paul, Clark, & Gordon, 2007). A more recent study (Fjell, Sneve, Grydeland, Storsve, & Walhovd, 2017) confirmed that the WM integrity overall, as well as individual tracts, correlates with EF. Specifically, WM integrity of the inferior longitudinal fasciculus (ILF) and that of the temporal part of the superior longitudinal fasciculus (SLF) were found to be most significantly correlated with the performance in the Stroop test, a common measure of EF. However, there have also been findings of strong functional connectivity existing between areas with apparently low or no structural connectivity (Cunningham, Tomasi, & Volkow, 2017).

In this study, we aimed to uncover and evaluate the WM tracts that underlie the structural connectivity between these functionally‐defined ECN nodes. This has been facilitated by the recent development of multi‐shell and high angular resolution dMRI sequences that allow improved description of white matter anatomy (Callaghan et al., 2018). Using a large, well‐described cohort, we applied high‐angular resolution dMRI to catalogue the WM structural network of the ECN, and to test explicitly how this relates to EF performance.

## 2 METHODS

### 2.1 Participants

We used data from 140 healthy participants from the Chronic Diseases Connectome Project (CDCP). Participants were recruited from the public using recruitment flyers placed at the University of Sydney, community‐based clubs, and online advertisements. Participants were over 18 years of age and free of neurological and cardiac disease, diabetes, renal impairment or MRI contraindication. They gave informed consent and the study had ethical approval from the Adventist Healthcare Limited Human Research Ethics Committee (2017–048) and the Macquarie University Medical Sciences Human Research Ethics Committee (5201500943). All experiments were performed in accordance with the relevant guidelines and regulations.

The demographic and psychometric characteristics of the cohort (*N* = 140) are summarised in Table 1. The cohort was representative of a normal population, with a WebNeuro's standard 10 and normalised EF scores centred at the population mean (Paul et al., 2005). The average age was 41.2 years (*SD* 15.3), from 18 to 79 years, with a spread concentrated across 3 decades (IQR: 28–53 years), and a slight predominance of females.

**Table 1:** Demographics and clinical measures summary

Demographics	N	%
Number	140	100
No. of females	82	58.6
	Mean	*SD*
Age (years)	41.17	15.34
Years of education	15.52	2.94
WebNeuro		
WebNeuro executive (standard 10)	5.94	1.95
WebNeuro executive (normalised)	0.14	0.95

### 2.2 Image acquisition

Diffusion MRI and structural T1‐weighted sagittal 3D SPGR MRI data were acquired at Macquarie Medical Imaging at Macquarie University Hospital (Sydney, Australia) as previously described (Grieve et al., 2013). Acquisition was performed using a 3‐Tesla GE Discovery MR750w MRI scanner (General Electric Healthcare, Milwaukee, Wisconsin) running DV25.1 software and using a 32‐channel Nova head coil. A contiguous AC‐PC aligned sagittal MPRAGE PROMO T1‐weighted image was acquired using the following parameters: TR = 8.39 ms, TE = 3.17 ms, TI = 900 ms, flip angle = 8°, matrix = 256 × 256, 198 slices, and 1 mm isotropic voxels. A multi‐shell multi‐band (factor = 3) diffusion pulse sequence was used for dMRI with a phase offset applied to each multi‐band component. We also acquired a *b* = 0 volume with reversed phase‐encoding for distortion and eddy‐current correction. Each dMRI dataset comprised 140 volumes of unique gradient directions (25 volumes at *b* = 700 s/mm^2^, 40 volumes at *b* = 1000 s/mm^2^, 75 volumes at *b* = 2,800 s/mm^2^) and eight interleaved *b* = 0 volumes, acquired with TR = 3,245 ms, TE = 100 ms, flip angle = 90°, 128 × 128 acquisition matrix, 66 slices, 2 mm isotropic voxels and FOV = 240 mm.

### 2.3 Neuropsychological assessment

Neurocognitive testing was performed for all subjects within a week of the MRI scan, using the “WebNeuro” standardised computer‐based battery of cognitive tests. The WebNeuro battery consists of a series of 12 tests and takes 30–40 min to complete (Silverstein et al., 2007). Participants were presented with instructions on screen prior to each of the tests with a researcher present to aid where necessary. The tests have been validated against pen‐and‐paper tests (Paul et al., 2005) and have sound test–retest reliability (Williams et al., 2005). This battery reports on four overall cognitive markers: thinking, emotion, feeling and self‐regulation each encompassing several variables (14 in total) obtained from one or multiple tasks. Thinking consisted of response speed, impulsivity, attention and concentration, information processing efficiency, memory and executive function; feeling consisted of depressed mood, anxiety and stress; emotion consisted of emotion identification and emotion bias; and self‐regulation included negativity bias, emotional resilience and social skills. Participants' height, weight, blood pressure, and pulse were recorded on the day at the testing site.

Specifically, the EF performance of the subjects was assessed by the Maze test, Switch Attention, Verbal Interference, and Go‐No‐Go tasks in the Webneuro battery (Silverstein et al., 2007). In our analysis, we derived the EF composite score from the raw subscores of these EF‐related tasks, using factor analysis accounting for the maximum variance (varimax) amongst raw subscore data. For the Maze test, we included the number of trials completed, completion time, path learning time, overrun errors, and total errors; for the Switch Attention, we included the completion time (digits and letters), average connection time (digits and letters), and number of errors; for the Verbal Interference, we included the errors and reaction time for congruent and non‐congruent stimuli, and for Go‐NoGo we included the reaction time, the variability of reaction time and false miss errors on Go, the false alarm errors on NoGo, as well as total errors.

### 2.4 Image processing

The structural T1‐weighted MRI images were segmented into white matter (WM), grey matter (GM) and cerebrospinal fluid (CSF) and the total intracranial volume (TIV) was estimated using FreeSurfer software version 6 (Fischl, 2012). The subcortical GM structures were segmented using a model‐based method implemented by FSL's FIRST tool (Patenaude, Smith, Kennedy, & Jenkinson, 2011). The T1‐weighted images were linearly registered to *b* = 0 images of the dMRI data (Jenkinson, Bannister, Brady, & Smith, 2002), and the anatomical labels were also transformed to the dMRI space.

With the right ECN (RECN) and the left ECN (LECN), each composed of six subregions, the atlas of ECN nodes defined on the standard MNI space (Shirer et al., 2012) were obtained from Stanford's Functional Imaging in Neuropsychiatric Disorders lab (http://findlab.stanford.edu/functional_ROIs.html). The ECN node labels were first transformed into T1‐weighted image's native space by linear registration followed by non‐linear registration using FSL's FNIRT tool and were then aligned to the diffusion images using the transformation obtained from the linear registration between T1‐weighted and diffusion images.

The dMRI data were corrected for eddy current induced distortion and motion due to involuntary head movement during the acquisition (J. L. R. Andersson & Sotiropoulos, 2016) with the b‐matrix rotated accordingly to compensate for the rotation component in the motion (Leemans & Jones, 2009). The susceptibility‐induced off‐resonance field in the diffusion images was estimated from data collected with reversed phase‐encode blips, resulting in pairs of images with distortions going in opposite directions (J. L. Andersson, Skare, & Ashburner, 2003) as implemented in FSL (S. M. Smith et al., 2004). MSMT‐CSD (Jeurissen, Tournier, Dhollander, Connelly, & Sijbers, 2014) was used to estimate the distribution of the fibre orientation distribution (FOD) in each voxel. This was performed using the MRTrix3 package (Brain Research Institute, Melbourne, Australia, http://www.mrtrix.org/) (Tournier, Calamante, & Connelly, 2012).

### 2.5 Tractography and connectomics

We performed Anatomically‐Constrained Tractography (ACT) (R. E. Smith, Tournier, Calamante, & Connelly, 2012) on the FODs estimated from the dMRI, informed by the segmentation of WM, cortical GM, subcortical GM, and CSF on the structural MRI. A total of 10 million streamlines were generated for each subject, and each streamline was assigned a weight using the SIFT2 algorithm (R. E. Smith, Tournier, Calamante, & Connelly, 2015) such that the weighted distribution of tracts in the generated tractogram is proportional to the estimated FOD. The total SIFT2 weight was normalised, such that for each subject the sum of SIFT2 weights of tracts in the whole tractogram was 10 million.

The connectome of each subject over the ECN was computed with the normalised SIFT2 weights, representing cross‐sectional area of each tracts as the measure of connection strength. For a given pair of nodes in the network, the connection strength was computed as the sum SIFT2 weights of all tracts connecting these two nodes. A fractional anisotropy (FA)‐based connectome, which used the average FA along all tracts connecting two nodes as the connectivity measure, was also computed.

### 2.6 Statistical analysis

In our analysis, we performed a univariate analysis of correlation between EF and connectivity followed by a network‐based analysis (NBS) (Zalesky, Fornito, & Bullmore, 2010) over ECN. These two analyses furnished evidences from different perspectives: the univariate analysis showing the relationship between individual connections and EF, and the NBS demonstrating the demonstrating the significance of ECN to EF. The agreement of these two analyses would show that the network‐wide association of EF to ECN is supported by the measurements at the level of individual tracts.

We carried out descriptive analysis of the ECN and EF performance, using generalised linear model (GLM) to evaluate the correlation between the EF and connectome as measured by connection strength and average FA. For a given connection between two nodes, the GLM modelled the EF score derived from WebNeuro as the dependent variable, and the connection strength or tract‐average FA as independent variable, with subject's age, sex, years of education, and TIV included as covariables (Hanggi, Fovenyi, Liem, Meyer, & Jancke, 2014; Jancke, Merillat, Liem, & Hanggi, 2015). We calculated the effect size in terms of Δ*R* ^2^, namely the difference in *R* ^2^ comparing models including and excluding the connectivity measurements as an independent variable. The statistical significance of the relationship between ECN connectivity and EF performance in terms of *p*‐value was also evaluated.

We excluded from the analysis any connections with less than 1% of the total SIFT2 weight in the ECN network, in order to include only connections with a substantial strength, thus reducing the effect of multiple comparisons.

NBS was used to evaluate the statistical significance of the correlation between EF performance and WM connectivity within the structural network and ECN. In NBS analysis, the connectome variables, namely the connection strength and tract‐average FA were used as dependent variable in GLM, with the EF score as the independent variable, and subject's age, sex, years of education, and TIV as covariables. The statistical significances of topological clusters were determined via permutation testing and corrected for the family‐wise error. In NBS, as in the univariate analysis, the connections with strength less than 1% of the total connectivity in ECN were also excluded.

### 2.7 Exploratory investigation of ECN anatomy

Based on structural connections between ECN nodes, we further investigated connectivity between the anatomical substructures of ECN nodes parcellated by Brainnetome atlas (Fan et al., 2016). For each component in the structural network identified by NBS, we computed the connectivities between the Brainnetome subdivisions, and evaluated their correlation with the EFs by a GLM.

## 3 RESULTS

A linear regression analysis on the EF composite score, which we calculated using factor analysis is shown in Table 2. The results showed strong correlations with age and years of education. The EF performance deteriorates significantly with age, and subjects with more years of education showed higher EF performance.

**Table 2:** Regression analysis of executive function (EF) composite score

	Regression coefficient (95% CI)	Standardised *β*	Effect size Δ*R* ^2^	*t*‐statistic	*p*
Age	−0.155 (−0.202 to −0.107)	−.47	.203	−6.39	<10^–8^*
Gender	1.48 (−0.34 to 3.25)	.14	.013	1.61	.20
Years of education	0.381 (0.128 to 0.634)	.22	.044	2.98	.003*
Total intracranial volume (TIV, mm^3^)	2.86 (−3.06 to 8.78) x10^−6^	.09	.004	0.95	.34

* *p* < .05.

### 3.1 Structural connectivity networks

**Figure 1:**
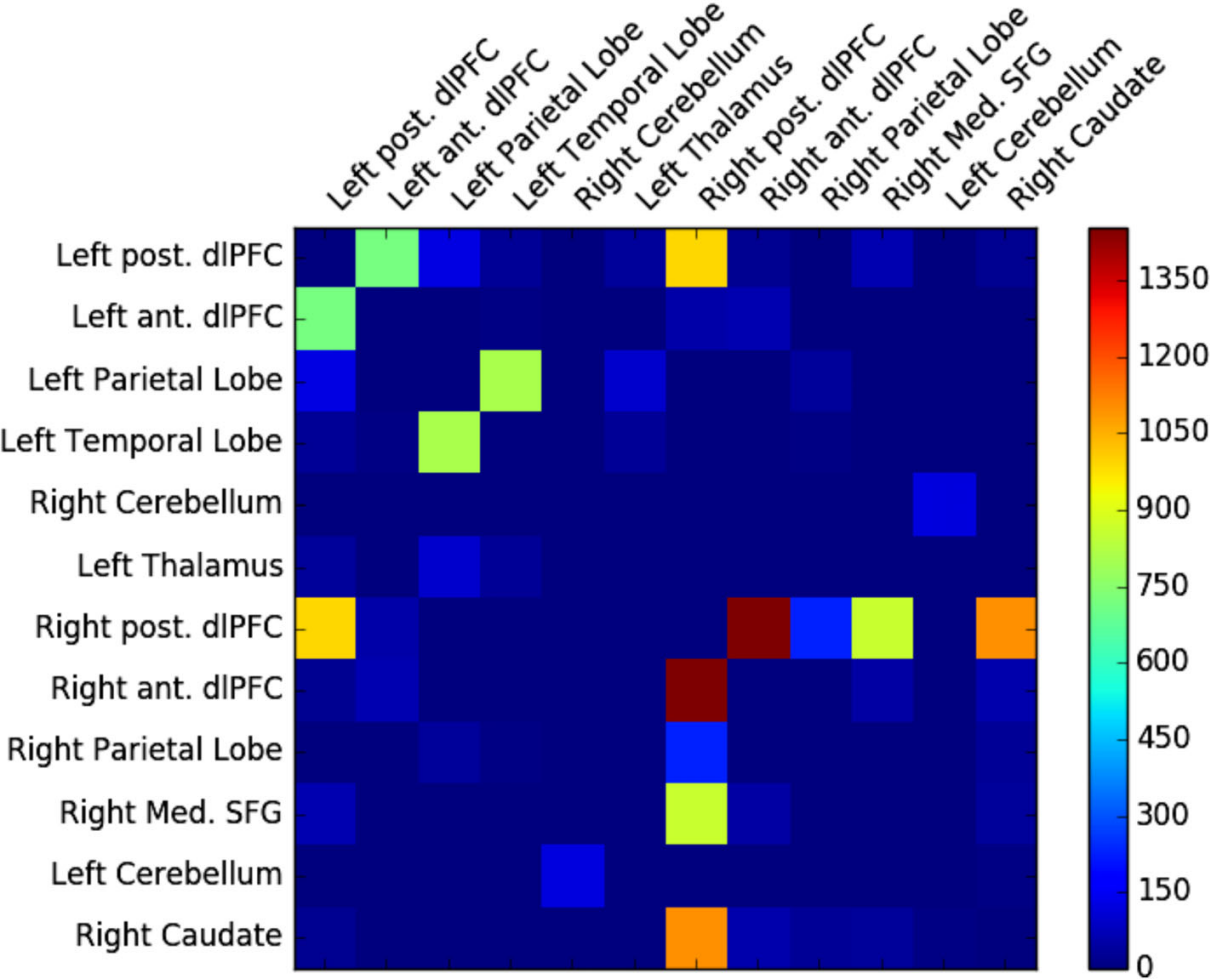
Average connectivity (*N* = 140) in the functionally defined Executive Control Network (ECN). dlPFC: dorsolateral prefrontal cortex; SFG: superior frontal gyrus

Figure 1 shows the average structural connectivity matrix across the ECN. Table 3 summarises the individual connection strengths above a threshold of 1% of total connections. The four dominant structural connections were: (a) the right orbitofrontal to the right dorsolateral prefrontal (R anterior dlPFC –R posterior dlPFC; FA 0.44; connection strength, CS: 1455.2 +/− 631.4); (b) the right dorsolateral prefrontal to caudate (R dlPFC–R Caudate; FA 0.48; connection strength, CS: 1101.1 +/− 733.5); (c) the left middle frontal gyrus to right dorsolateral prefrontal (L dlPFC–R dlPFC; FA 0.57; connection strength, CS: 992.1 +/− 537.8); and (d) the right superior frontal gyrus to the right dorsolateral prefrontal (R medial SFG–R dlPFC; FA 0.43; connection strength, CS: 863.6 +/− 413.7). These 4 connections accounted for 61.8% of the total connection strength (R anterior dlPFC–R posterior dlPFC = 20.4%; R post. dlPFC–R Caudate = 15.4%; L posterior dlPFC–R posterior dlPFC = 13.9%; R medial SFG–R dlPFC =12.1%).

**Table 3:** Univariate relationship between EF performance and the top 10 tracts in ECN ranked by connection strength

Tract	% of total connection strength in ECN	Mean FA of the tract	Standardised *β* of EF term	Effect size Δ*R* ^2^	*t*‐statistic (*p*‐value) of EF term
R ant. dlPFC–R post. dlPFC	20.37	0.44	.09	0.007	1.21 (.23)
R caudate–R post. dlPFC	15.41	0.48	.12	0.012	1.56 (.12)
L post. dlPFC–R post. dlPFC	13.88	0.57	.17^a^	0.027	2.35 (.02)*
R med. SFG–R post. dlPFC	12.09	0.43	.05	0.002	0.61 (.54)
L temp.–L parietal	11.30	0.55	.15^a^	0.021	2.05 (.04)*
L ant. dlPFC–L post. dlPFC	10.04	0.46	.02	0.001	0.34 (.74)
R parietal–R post. dlPFC	3.21	0.52	.15^a^	0.023	2.16 (.03)*
L parietal–L post. dlPFC	1.78	0.51	.12	0.013	1.65 (.10)
L Cb.–R Cb.	1.71	0.31	.02	<0.001	0.21 (.83)
L thalamus–L parietal	1.42	0.49	.09	0.008	1.24 (.21)

a denotes significant term in regression model.

Abbreviations: ant., anterior; Cb, cerebellum; dlPFC, dorsolateral prefrontal cortex; L, left; med., medial; post., posterior; R, right; SFG, superior frontal gyrus.

**Table 4:** ECN components correlating with EF based on NBS

Tract	Correlation between the connection strength and EF	Effect size Δ*R* ^2^	% of total connection strength in ECN	NBS FWE corrected *p* value
Connectivity measured by SIFT2				
L temp. Lobe–L par. Lobe–L post. dlPFC – R post. dlPFC–R parietal lobe – R caudate	0.284	0.054	45.6	0.003
Connectivity measured by FA				
R parietal–R post. dlPFC–R ant. dlPFC–R caudate	0.198	0.026	39.0	0.016

Additional strong connections were seen involving the left anterior dlPFC, bilateral cerebellum, and right parietal lobe, which together accounted for an additional 31.6% of total connections (see connections ranked 5–10 in Table 3).

### 3.2 Univariate relationship between ECN connection strengths and EF performance

**Figure 2:**
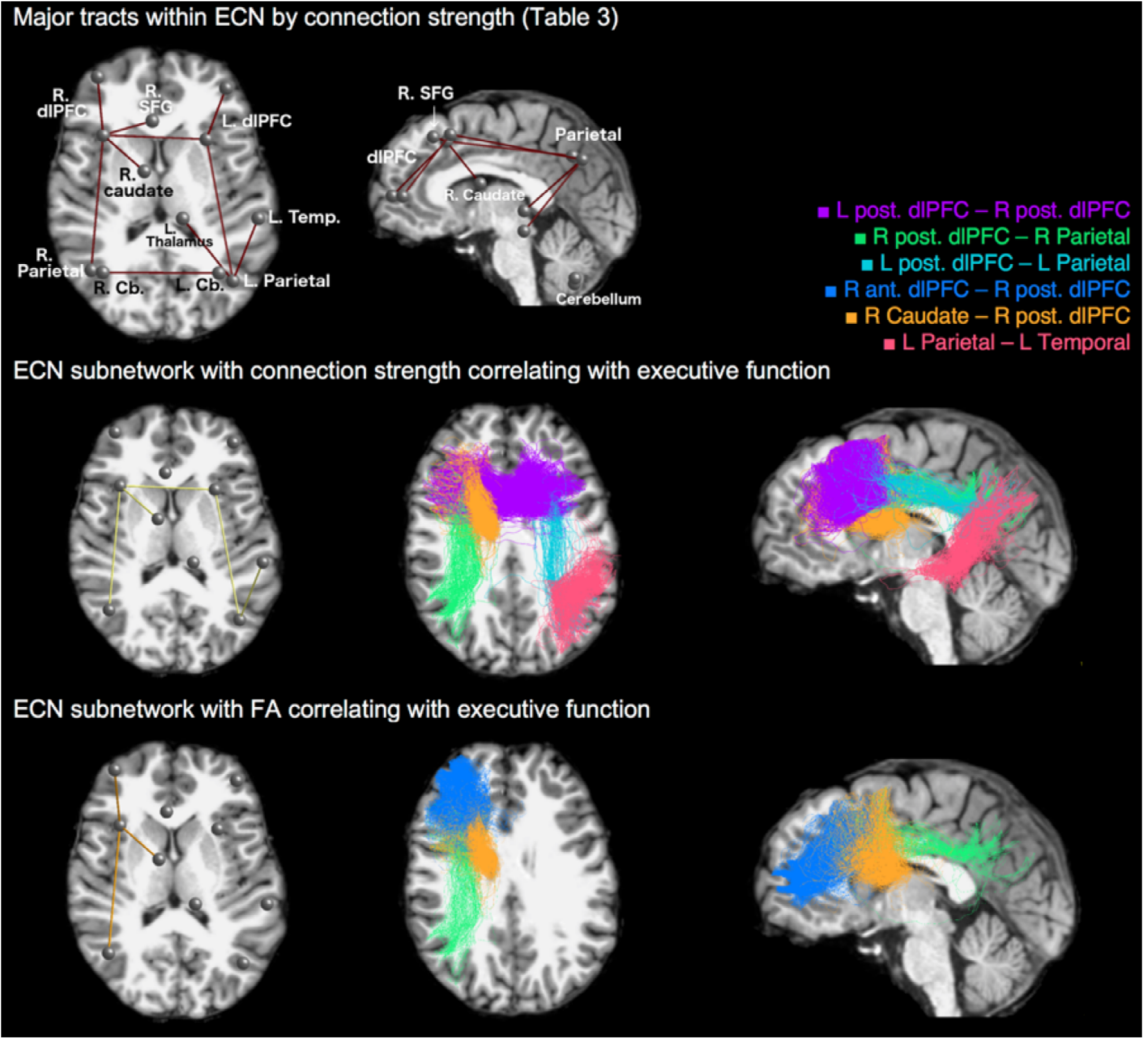
The structural subnetworks in ECN correlated with EF performance. Top row: the major white matter tracts in ECN listed in Table 3. Middle row: the ECN subnetwork with significant positive correlation between EF and structural connectivity measured by connection strength in NBS analysis with t‐statistic threshold *t* = 1.5. From left to right: the ECN nodes and the connections that form the subnetworks correlating with EF, axial and sagittal views of sample tractograms of the ECN subnetworks identified in Table 4: R Parietal–R Caudate–R post. dlPFC–L post. dlPFC–L Parietal–L Temporal. Bottom row: the subnetwork showing significant correlation between EF and FA‐based connectivity in NBS analysis with threshold *t* = 1.5. R Parietal–R post. dlPFC–R ant. dlPFC–R Caudate. ECN, executive control network; EF, executive function; dlPFC, dorsolateral prefrontal cortex

A univariate correlation between performance and connection strength controlling for subject's age, sex, and years of education was performed in order to evaluate the relationships between each component of the ECN. The analysis was only performed for connections with a total connection strength >1% of total inter‐node connections and is presented in Table 3, as well as in Figure 2. An exploratory analysis was also performed across all the ROIs from the Desikan–Killiany atlas (Supplementary Table S1). Connections between the superior frontal and the rostral middle frontal in both hemispheres showed correlations of similar strength to the ECN connections.

### 3.3 Network based statistics

The results of NBS analysis of the relationship between ECN connectivity (SIFT2 weights) and EF performance are shown in Figure 2. With the t‐statistic threshold at *t* = 1.5, NBS reveals a single dominant component in ECN within which connection strength positively correlated with EF performance. This subnetwork connects the parietal lobe, dlPFC, caudate in the right hemisphere and the left dlPFC, parietal and temporal lobes, with effect size Δ*R* ^2^ = 0.054 and FWE corrected *p* = .003. The correlation coefficient between total connection strength within this subnetwork and EF score is 0.284 (*p* = .005), controlling for age, gender years of education, and TIV.

The NBS analysis of the FA‐based structural connectivity identified at threshold *t* = 1.5 a subnetwork in the right hemisphere correlating with EF. It consists of fronto‐parietal connection between the parietal lobe and dlPFC, and a fronto‐striatal connection between right posterior dlPFC and right caudate (FWE corrected *p* = .019). The correlation coefficient between the connection strength of this subnetwork and EF score corrected for age, gender, education and TIV was 0.198 (*p* = .05).

No significant negative relationship between EF performance and structural connectivity (either connection strength or FA) in ECN components was found in NBS.

### 3.4 Exploratory analysis of ECN anatomy

The results of our exploratory analysis of ECN substructures were shown in Table 5, in which the major connections between anatomical Brainnetome defined nodes were evaluated for connection strength only. The results confirmed that the univariate correlations with EF of connection strength between anatomical Brainnetome nodes were consistent with those between ECN nodes (Table 3). No single univariate correlation of region‐to‐region connection strength and EF approached that of the whole sub‐network (*β* = .284). The subregions from the Brainnetome did reveal some considerable regional heterogeneity, however, with the strongest functional correlations typically being more than 100% stronger than the weakest within each node. The strongest univariate correlation with EF performance in the L Parietal–L posterior dlPFC node was between the inferior frontal junction and caudal area 40 (*β* = .18, Δ*R* ^2^ = 0.026, versus *β* = .09, Δ*R* ^2^ = 0.006 for the weakest connection within these regions). Similar heterogeneity was seen in the L posterior dlPFC–R posterior dlPFC node (*β* = .17, Δ*R* ^2^ = 0.028 for the L dorsolateral area 8–R ventrolateral area 8, versus *β* = .10, Δ*R* ^2^ = 0.010 for the weakest connection within this), and to a lesser degree in the other nodes.

**Table 5:** The connection strength between Brainnetome nodes and EF within the ECN components identified by ECN

Tract	% of connection strength within ECN	Standardised *β* of EF term	Effect size Δ*R* ^2^	*t*‐statistic (*p*‐value) of EF term
R anterior dlPFC–R posterior dlPFC (correlation with EF *β* = .09)				
Ventral area 9/46–ventral area 9/46	3.69	.10	0.010	1.21 (.23)
Ventral area 9/46–ventrolateral area 8	3.01	−.02	< 0.001	−0.27 (.79)
Ventrolateral area 8–area 46	2.62	−.02	< 0.001	−0.26 (.80)
Ventral area 9/46–area 46	2.12	.09	0.007	1.04 (.30)
Dorsal area 9/46–area 46	1.45	−.04	0.002	0.49 (.63)
R caudate–R posterior dlPFC (correlation with EF *β* = .12)				
Ventrolateral area 8–dorsal caudate	5.11	.18*	0.028	2.06 (.04)*
Dorsolateral area 8–dorsal caudate	2.52	.16	0.021	1.79 (.08)
Ventrolateral area 6–dorsal caudate	1.94	.16	0.023	1.87 (.06)
Dorsal area 9/46–dorsal caudate	1.87	.19*	0.026	1.99 (.05)*
Inferior frontal junction–Dorsal caudate	0.81	.10	0.009	1.17 (.25)
L posterior dlPFC–R posterior dlPFC (correlation with EF *β* = .17)				
L dorsolateral area 8–R dorsolateral area 8	1.94	.10	0.010	1.21 (.23)
L ventrolateral area 8–R dorsolateral area 8	1.84	.10	0.010	1.19 (.24)
L ventrolateral area 8–R ventrolateral area 8	1.77	.13	0.014	1.46 (.15)
L dorsolateral area 8–R ventrolateral area 8	1.73	.17*	0.028	2.06 (.04)*
L ventrolateral area 8–R dorsal area 9/46	1.15	.14	0.018	1.63 (.11)
L temp.–L parietal (correlation with EF *β* = .15)				
Caudal lateral area 20–caudal area 40 (PFm)	2.10	.01	< 0.001	0.17 (.87)
Caudal lateral area 20–rostrodorsal area 39 (Hip3)	2.04	.07	0.005	0.84 (.40)
Caudal lateral area 20–rostroventral area 39 (PGa)	1.86	.05	0.003	0.61 (.54)
Caudal area 21–Caudal area 40 (PFm)	0.96	.05	0.002	0.59 (.56)
Caudal area 21–rostrodorsal area 39 (Hip3)	0.89	.06	0.003	0.69 (.49)
L anterior dlPFC–L posterior dlPFC (correlation with EF *β* = .02)				
Ventral area 9/46–ventrolateral area 8	1.53	.09	0.007	1.04 (.30)
Ventrolateral area 8–rostral area 45	0.94	.03	0.001	0.40 (.69)
Dorsolateral area 8–ventral area 9/46	0.89	.04	0.002	0.51 (.62)
Ventrolateral area 8–inferior frontal sulcus	0.79	.02	< 0.001	0.20 (.84)
Dorsolateral area 8–rostral area 45	0.71	.04	0.001	0.43 (.67)
R parietal–R posterior dlPFC (correlation with EF *β* = .15)				
Ventrolateral area 8–caudal area 40 (PFm)	0.58	.07	0.004	0.80 (.42)
Ventrolateral area 6–caudal area 40 (PFm)	0.30	.07	0.005	0.86 (.39)
Ventrolateral area 8–rostrodorsal area 39 (Hip3)	0.29	.14	0.018	1.63 (.11)
Ventrolateral area 8–rostrodorsal area 40 (PFt)	0.24	.02	< 0.001	0.19 (.85)
Ventrolateral area 6–rostrodorsal area 39 (Hip3)	0.20	.08	0.006	0.97 (.33)
L parietal–L posterior dlPFC (correlation with EF *β* = .12)				
Ventrolateral area 8–rostrodorsal area 39 (Hip3)	0.32	.13	0.012	1.33 (.19)
Ventrolateral area 8–caudal area 40 (PFm)	0.24	.09	0.006	0.93 (.35)
Inferior frontal junction–rostrodorsal area 39 (Hip3)	0.16	.15	0.019	1.68 (.10)
Inferior frontal junction–caudal area 40 (PFm)	0.15	.18*	0.026	1.98 (.05)*
Ventrolateral area 6–rostrodorsal area 39 (Hip3)	0.15	.10	0.009	1.12 (.27)
L thalamus–L parietal (correlation with EF *β* = .09)				
Rostrodorsal area 39 (Hip3)–posterior parietal thalamus	0.49	.09	0.007	1.00 (.31)
Caudal area 40 (PFm)–posterior parietal thalamus	0.33	.04	0.001	0.45 (.65)
Rostroventral area 39 (PGa)–posterior parietal thalamus	0.23	.08	0.005	0.89 (.37)
Rostrodorsal area 39 (Hip3)–occipital thalamus	0.07	.10	0.010	1.21 (.23)
Caudal area 39 (PGp)–posterior parietal thalamus	0.07	.10	0.010	1.20 (.23)

* *p* < .05.

## 4 DISCUSSION

Our study reveals the presence of a “structural core” in the ECN, providing convergent evidence linking components of the functionally‐defined ECN with structural network strength. This network was defined by both connection strength and integrity (as measured by FA), and involved the bilateral dlPFC, fronto‐parietal network, and the right caudate. In a cognitively normal cohort, we that found that the connection strength of this network significantly correlated with the overall performance of EF‐related tasks. We suggest that this “structural core” network may represent the static architecture from which the dynamic functional connectivity underlying EF emerges (Park & Friston, 2013).

We found that the structural connection between bilateral prefrontal cortices formed a key part of the network that is highly correlated with EF performance.

The dominant components of the ECN “structural core” are the “fronto‐parietal network” between the dlPFC and the parietal nodes, with a correlation of 0.16 (right) and 0.13 (left) standardised *β* with EF. This agrees with previous evidence that WM tracts connecting frontal and parietal lobes, mainly via SLF, are associated with better EF performance (Gallen, Turner, Adnan, & D'Esposito, 2016; Sasson, Doniger, Pasternak, Tarrasch, & Assaf, 2013; Smolker, Depue, Reineberg, Orr, & Banich, 2015; Smolker, Friedman, Hewitt, & Banich, 2018; Zhang et al., 2019). Others have identified WM tracts connecting the frontal and parietal lobes through the cingulum, whose WM integrity was also previously reported to be associated with EF performance (Bettcher et al., 2016).

The left and right hemispheres of the brain are connected by the WM of the corpus callosum, which is involved in the shifting and inhibition aspects of EF (Bettcher et al., 2016). The genu of the corpus callosum interconnecting the frontal lobes and the splenium‐parietal connections in the right hemisphere have both been found to mediate the ageing effect on task switching performance in EF (Madden, Bennett, & Song, 2009). Our analysis using the Brainnetome atlas allowed greater delineation of the anatomical specificity of the structural core, showing that the connection strength between left and right Brodmann area 8 has a stronger association with EF than the connection between larger ECN dlPFC nodes.

Fronto‐striatal connections between right dlPFC and caudate are associated with EF performance in the FA based network. The Brainnetome analysis showed this was concentrated between the Brodmann area 8 and the dorsal part of caudate. The microstructural characteristics of fronto‐striatal WM have been reported to correlate with EF in a younger population (Chiang, Chen, Shang, Tseng, & Gau, 2016), and our results generalise this observation to a broader age range. The caudate is known to be involved in working memory tasks, especially during the encoding phase of these tasks (Moore, Li, Tyner, Hu, & Crosson, 2013).

Our results show that disconnection within the “structural core” of the ECN network is associated with poorer EF performance, independent of age. Prior investigators have shown changes in EF performance with age may be more strongly predicted by changes in brain structure than by functional connectivity (Fjell et al., 2017). Fjell and colleagues demonstrated WM volume changes and brain connectivity together explained nearly half of the decline in EF, whereas functional connectivity alone explained nearly none. Our results extend these observations by revealing the specific anatomical detail of the circuits that may be most important to this process of “disconnection” with age. In our tractography‐based analysis, we normalised the total number of connections across the cohort, thus controlling for the effect of decline of global connectivity with age. The normalised total structural connectivity within ECN did not show significant decline over age (*β* = −.05, *p* = .60). Thus, the structural connectivity in our analysis measured network disconnections independent of the global effect from ageing.

In our analysis of structural connectivity in relation to EF, we used consensus data derived from functional MRI to guide our investigation. This prior data enabled us to narrow the scope of tractography based analysis of structural network to a few nodes of ECN that are consistently shown to be involved in EF‐related activities. An exploratory analysis performed for the whole brain, without specifically targeting these nodes, did not reveal any further relationships between the strength of connectivity and EF performance, but did reveal strong correlations with general fluid cognition. While not definitive, this supports the view that, for the EF component, the relationship between structural features and function is quite specific to a subnetwork of connections (Madden et al., 2017). This may, however, only be true in the absence of severe dysfunction: previous work has shown correlation between global WM in dMRI and EF performance in a cohort with cognitive decline, but not amongst the normal subjects (Ohlhauser, Parker, Smart, & Gawryluk, 2019).

We measured the structural connectomes on a high angular resolution dMRI dataset with 140‐gradient directions with higher accuracy compared to the conventional 64‐direction DTI protocol. The structural connectome of normalised connection strength is shown to have higher reliability (Prckovska et al., 2016) than DTI‐based measurements such as TBSS (Madhyastha et al., 2014), and using high angular resolution dMRI has shown better longitudinal consistency (Prckovska et al., 2016). We previously showed that these high angular resolution datasets provide an improved delineation of key white matter pathways (Callaghan et al., 2018) including the most detailed model to date of the hippocampal connectome (Maller et al., 2019).

In our analysis, we measured the EF by a single composite score aggregating subjects' performance on various EF‐related tasks, namely Maze test, switch of attention, verbal interference, and Go‐NoGo. This may mask the different components of EF measured by these tasks, including abilities to updating working memory, switching between mental sets, and inhibition of prepotent response (Miyake et al., 2000). There are connections between specific subsets of EF related to distinct circuitries (Tekin & Cummings, 2002) which are not investigated in this work.

To conclude, we investigated the structural WM network underlying the functionally defined ECN using tractography‐based analysis on high angular resolution dMRI data. We identified a WM network comprising the fronto‐parietal SLF tracts between dlPFC and the parietal lobe, the corpus callosum between bilateral dlPFC, and the fronto‐striatal connection between right dlPFC and caudate. This formed a structural network at the core of the functional ECN, and the structural connectivity of this network significantly correlated with EF performance. Alongside previous studies, there is convergent evidence for this structural core subnetwork of the functional ECN that may be crucial to our future understanding of higher cognitive function.

## CONFLICT OF INTERESTS

SB acknowledges speaking honoraria from Novartis, F. Hoffmann La Roche and the Alzheimer's Association not related to the work presented here.

## Supporting information


**Table S1** Major tracts from whole‐brain structural network (defined by Desikan‐Killiani atlas) with fibre connectivity, FA, correlation EF performance
**Table S2**: Subnetwork components of the whole‐brain network correlating with EF based on NBS

## Data Availability

The data that support the findings of this study are available from the corresponding author upon reasonable request.
