# Supplementary material for: Structural core of the executive control network: A high angular resolution diffusion MRI study
Source: Hum Brain Mapp. 2019 Nov 25;41(5):1226–36. doi: 10.1002/hbm.24870 (PMC7267982; doi:10.1002/hbm.24870)
Supplement: Supplementary file 1 — Table S1 Major tracts from whole‐brain structural network (defined by Desikan‐Killiani atlas) with fibre connectivity, FA, correlation EF performance Table S2: Subnetwork components of the whole‐brain network correlating with EF based on NBS [file HBM-41-1226-s001.docx]

**SUPPLEMENTARY MATERIAL**

**Supplementary Table 1**: Major tracts from whole-brain structural network (defined by Desikan-Killiani atlas) with fibre connectivity, FA, correlation EF performance

| Connection | % of total connections | Mean FA | t-statistic | p-value |
| --- | --- | --- | --- | --- |
| R Pericalcarine – R Lat Occipital | 1.77 | 0.37 | 0.28 | 0.78 |
| L Sup. Frontal – L Rostral Mid. Front. | 1.66 | 0.43 | -0.29 | 0.77 |
| R Sup Parietal – R Precuneus | 1.48 | 0.44 | 1.97 | 0.051 |
| L Pericalcarine – L Lat. Occipital | 1.47 | 0.36 | -0.08 | 0.94 |
| R Sup Frontal – R Rostral Mid. Front. | 1.44 | 0.42 | 1.61 | 0.11 |
| L Mid Temporal – L Inf. Temporal | 1.23 | 0.39 | 1.19 | 0.24 |
| R Mid Temporal – R Inf Temporal | 1.23 | 0.40 | -0.59 | 0.56 |
| L Sup Parietal – L Precuneus | 1.12 | 0.44 | 2.71 | 0.0077 |
| L Lat Occipital – L Inf Parietal | 1.06 | 0.43 | -1.07 | 0.28 |
| R Pericalcarine – R Cuneus | 1.04 | 0.36 | -0.78 | 0.43 |

**Supplementary Table 2**: Subnetwork components of the whole-brain network correlating with EF based on NBS

| Network nodes | % of total connections | NBS FWE corrected p |
| --- | --- | --- |
| R Pars Orb, R Sup Frontal, R Rostral ACC, R Precentral, R Pallidum, , R Frontal Pole, R Rostral Mid Frontal, R Med OFC, L Pars Orb, L Precentral, L Insular, L Lat OFC, L Med OFC, L Rostral ACC, L Rostral Mid Frontal, L Sup Frontal, L Frontal Pole, L Pallidum, L Hippocampus | 7.62% | 0.021 |
| R Postcentral, R Post Cingulate, R Precuneus, R Sup Parietal, R Putamen, R Sup Temporal, R Supra Marginal, R Transverse Temporal, R Insula, R Cuneus, R Inf Parietal, R Inf Temporal, R Lat Occipital, R Lat OFC, R Lingual, R Mid Temporal | 10.34 | 0.049 |
